# ‘Remember, You Are Not Alone’: A Qualitative Internet‐Mediated Study of Anxiety Among Adolescents on Reddit

**DOI:** 10.1111/inm.70183

**Published:** 2025-11-28

**Authors:** Greta Kaluzeviciute, Joshua P. I. Moreton, Jessica E. Jackson

**Affiliations:** ^1^ Department of Clinical Psychology, Institute of Psychology, Faculty of Philosophy Vilnius University Vilnius Lithuania; ^2^ College of Health, Psychology and Social Care School of Psychology University of Derby Derby UK; ^3^ School of Health Sciences Faculty of Medicine and Health Sciences University of Nottingham Nottingham UK

**Keywords:** adolescent mental health, anxiety, internet‐mediated research, online interactions, reddit

## Abstract

Recent evidence indicates that social anxiety disorders are increasing alongside a growing sense of disconnection from communities. However, anxiety disorders are often overlooked or perceived as ‘less serious’ mental health issues. Consequently, young people may avoid formal psychological interventions or discussions with family members for fear of being misunderstood, increasingly turning instead to online spaces for support. This study aimed to explore how adolescents (aged 12–18) use Reddit to share their experiences of anxiety. A total of 105 posts, made between January 2021 and January 2022, were thematically analysed. Three themes were identified: Perceived barriers in seeking mental health support; Reddit as a last resort: motivators leading to anonymous posts; and ‘Remember you are not alone’: empathy and support through distance. The findings highlight adolescents' fear of stigma and rejection, as well as limited access to appropriate mental health services. For many, online help‐seeking provided opportunities to share distress, seek advice, and connect with empathetic peers. Understanding social media's role in adolescents' emotional regulation, identity, and peer support can inform empathetic, tailored care. Encouraging open dialogue about online experiences supports safe coping and strengthens therapeutic relationships. Professionals should help adolescents build digital literacy and consider the potential therapeutic value of weak online ties, while research on practicalities, safeguarding, and outcomes is needed before implementation.

## 1 Introduction

Anxiety is a commonly diagnosed mental health condition (Royal College of Psychiatrists 2022), yet its conceptualisation in literature has been complex and inconsistent, complicating research and diagnosis (Bailey and Andrews 2003). Broadly, anxiety can be described as a psychological state marked by excessive fear, avoidance behaviours, and anticipatory worry about encountering a feared object or situation (Siegel and Dickstein 2011). Diagnostic frameworks, such as the DSM‐5‐TR (American Psychiatric Association 2022), categorise various anxiety disorders based on the relationship between the individual and the anxiety source. These include agoraphobia, generalised anxiety disorder (GAD), panic disorder, social anxiety disorder (SAD), separation anxiety, and others. The distinction between anxiety as a general emotional state and as a clinical disorder is often blurred (Daniel‐Watanabe and Fletcher 2021). However, anxiety disorders are typically defined by their chronic nature and long‐term impact on functioning and well‐being, as opposed to more situational forms of anxiety. SAD, in particular, sits at the intersection of this conceptual divide. It involves extreme distress during social interactions, physical symptoms such as trembling or blushing, and avoidance behaviours (Jefferson 2001). While situational anxiety, like fear of public speaking, may not be clinically significant, SAD is often rooted in a deeper fear of negative evaluation, especially problematic during adolescence, when social and emotional development is critical (Jefferies and Ungar 2020). Other forms, like separation anxiety and GAD, are frequently minimised, in part because they appear relatable and are often mistaken for normal life experiences (Denizet‐Lewis 2017). Despite this, these conditions can have serious implications and are often shaped by complex psychosocial factors such as gender, socioeconomic status, co‐occurring mental health conditions, substance misuse, and trauma. Further research is needed to explore both the underlying causes and diverse presentations of anxiety.

### 1.1 Anxiety in Adolescents

Anxiety disorders are highly prevalent among adolescents and are often considered a gateway to other mental health issues and distress (Legerstee et al. 2011; Howard 2018). Research suggests that phobic disorders in children and adolescents—particularly social phobia, agoraphobia, and specific phobia—are more common than reported in epidemiological studies (Bener et al. 2012). In recent years, there has also been an increase in social anxiety disorder (SAD) among young people, alongside a growing sense of disconnection from their communities (Office of National Statistics 2020). This trend became especially apparent during the COVID‐19 pandemic, which brought significant changes to young people's social and academic lives. During this period, 21.7% of 17 to 22‐year‐olds with a mental health concern reported not seeking help due to the pandemic (NHS Digital 2020). The crisis impacted not only social connections but also the ability to seek support through them. Many young people expressed fear of discussing their anxiety, often due to encountering anxiety‐related reminders and experiencing intense feelings of shame and guilt (McMillan et al. 2014). This presents barriers to accessing formal psychological support such as psychotherapy. General coping strategies for anxiety, burnout, and related conditions are well documented in psychological literature (Legerstee et al. 2011; Shin et al. 2014). These strategies include both cognitive and behavioural approaches, such as acceptance, positive reappraisal, planning, and positive refocusing (Garnefski et al. 2002). Cognitive coping, which involves managing emotional responses through mental effort, is particularly important in adolescents (Legerstee et al. 2011). Maladaptive strategies, such as self‐blame, rumination, and catastrophising, can worsen anxiety. However, qualitative research into adolescents' coping strategies remains limited, especially in relation to social media, which may itself act as a coping tool (Law et al. 2020; Naslund et al. 2020).

### 1.2 Adolescents in the Virtual Space: Help‐Seeking Behaviours on Reddit

People frequently seek help from professionals such as GPs, counsellors, and psychotherapists. However, these formal avenues can sometimes lack the empathy and emotional support needed by individuals experiencing physical or mental health difficulties (Aoun et al. 2018; Helgeson et al. 2001; Rabow et al. 1999; Wilson et al. 2010). As a result, many turn to informal support systems, such as friends, family, or even strangers (Collins et al. 2011; Wright and Rains 2013). Increasingly, individuals seek support online, where they can access experiential knowledge shared by others who face similar struggles (Wright and Miller 2010). Online communities allow for emotional disclosure in a safe and often anonymous space, helping to reduce stigma (Greene 2009; Steuber and Solomon 2011; Alqassim et al. 2019). Adolescents, in particular, are turning to digital spaces for emotional support (Pretorius et al. 2019). Social media platforms serve multiple mental health functions for young users, including venting emotions (Ophir 2017), sharing personal experiences (Vermeulen et al. 2018), and seeking connection with others (Meng et al. 2017). Support‐related behaviours are especially prominent among adolescent ‘transceivers’—those who both give and receive support online (Lipshits‐Braziler et al. 2022). As such, social media has become a crucial tool in understanding how adolescents experience and express mental health concerns (Dixon‐Ward and Chan 2022).

One platform central to this phenomenon is Reddit, which attracts around 52 million daily users, including a significant number of adolescents aged 13–18 (Lea et al. 2020). Reddit hosts topic‐specific communities called ‘subreddits,’ many of which focus on mental health issues such as anxiety and depression. These communities offer a semi‐anonymous space where users can share sensitive information, receive support, and avoid the emotional vulnerability often felt in face‐to‐face interactions (De Choudhury 2014). Research shows that many young people now prefer discussing mental health concerns online rather than with professionals or close contacts, due to the perceived objectivity and reduced emotional risk in digital environments (De Choudhury 2014; Dixon‐Ward and Chan 2022; Park and Conway 2018; Park et al. 2018; Northouse 1988; Wright and Rains 2013). This study aims to explore how adolescents use Reddit to share their experiences with anxiety, highlighting online help‐seeking behaviours, coping strategies, and the perceived impact of digital support on their well‐being.

The findings of this study will provide insight into young populations that are experiencing mental health distress but, for various reasons, are not seeking or are not able to seek formal mental health interventions or help from close family members. As such, our study will seek to provide a closer insight into a population that is typically underrepresented in traditional psychological research.

## 2 Methods

### 2.1 Study Outline

This study adopted an internet‐mediated research approach to facilitate the examination of typically underrepresented groups, such as adolescents actively using social media platforms to express and share their mental health experiences. This approach employs the internet as a medium through which information is gathered systematically and analysed (Hewson and Laurent 2008). In the context of our study, we conducted secondary internet research by assessing existing data (user posts) on Reddit. Therefore, this research did not directly engage with participants. Common ethical considerations to this approach often involve deliberations of what constitutes public or private domain in disembodied spaces. Reddit, a discussion platform open to the public, enforces strict rules against sharing personal or identifiable information and encourages the use of pseudonymous accounts. These features align with the requirements of our research study (Pappa et al. 2017). Furthermore, many subreddits are moderated communities, which means that posts are actively reviewed to avoid personal or inappropriate information.

In our study, we selected three different subreddits focusing directly on anxiety and/or mental health issues that involve experiences of anxiety. The selected subreddits, at the time of data extraction, had over 1000 members each. Before extracting posts from each subreddit, we sent a private message to moderators on the platform to inquire about the possibility of research and to inform them about our study aims and methodology.

### 2.2 Data Collection

The data collection process used the Baumgartner Reddit Corpus, a free, publicly available API, which has a track record of use in academic research (Medvedev et al. 2019). Baumgartner's search engine was used to search posts within specific timelines. We supplemented this method with direct (manual) searches of the relevant subreddits to reduce missing data (Gaffney and Matias 2018). Post‐extraction, the data cleaning commenced removing any identifiable information. Additional guidance concerning anonymising online data was followed (McDermott et al. 2015).

### 2.3 Analysis

The retrieved online posts were systematically organised to extract meaningful patterns across the dataset (Braun and Clarke 2006). Since this study aims to identify divergent idiosyncratic experiences pertaining to expressions of anxiety online, thematic analysis was deemed appropriate and has been used in similar studies (Dixon‐Ward and Chan 2022). Thematic analysis was carried out in the following order to identify the relevant themes: (i) Familiarisation, (ii) Generating initial codes, (iii) Searching for themes, (iv) Reviewing themes, and (v) Defining and naming themes (Braun and Clarke 2012).

Recognising the significance of researchers' thoughts, ideas, and emotions during the process of thematic analysis is crucial (Braun and Clarke 2006). In this study, we adopted a critical realist perspective (Sayer 1992) to help with this. While critical realism acknowledges the substantial social construct nature of our world (meaning we cannot separate our beliefs from our understanding of the world), it also promotes the development of realistic and causally meaningful interpretations for intricate social phenomena. As such, actively reflecting on how our social and linguistic practices influence and transform research findings and analytical methods is an integral part of a critical realist analysis.

The research was conducted by three individuals representing different areas of expertise: a psychotherapy researcher, a nurse researcher, and a social psychology researcher. The synergy of these diverse fields ensured a dynamic and iterative approach (Easton 2010), which prevented unquestioning acceptance of any single causal explanation, theme, or interpretation. This approach allowed researchers to critically evaluate and compare differing research findings.

The process of data triangulation was employed throughout the study to compare retrieved codes and themes as well as the quality of extracted passages. This process involves different researchers comparing their findings, perspectives, and methodological perspectives (Carter et al. 2014).

### 2.4 Study Sample

Data was extracted from posts written in English on moderated subreddits focusing on anxiety and mental health. Our study analyzed 105 posts made between January 2021 and January 2022 (this is to ensure that any personal information in the original post has been removed by subreddit moderators, as this can take some time). In addition, many posts have been removed (either by the users themselves or by the moderators), thus limiting the number of available posts for analysis. Each researcher analyzed available posts from a specific timeline (periods have been split in the following manner: January 2021–May 2021; June 2021–October 2021; November 2021–January 2022). In order to extract posts from select subreddits and timelines, researchers searched for posts and comments that include the words ‘anxiety’, ‘social anxiety’, ‘agitation’, ‘anxiousness’, ‘social‐phobias’, ‘panic attacks’, etc. Other mental health conditions that are often associated with anxiety have also been searched, e.g., ‘depression’, but they would need to be associated specifically with a form of anxiety disorder or a chronic feeling of anxiety to be extracted. The study targeted posts authored by individuals aged 12–18. Only posts that clearly indicated the author's age have been selected (see Table 1). To ensure that we include posts from our study sample, we employed Boolean operators to indicate both the searched term as well as the searched age (e.g., *Anxiety and 14*). All comments on the extracted posts were reviewed; comments that directly respond to the original post (OP) and offer relevant qualitative data in the context of our study (e.g., mental health advice, insight, shared experiences, reassurance, reaction to a mental health condition or symptom) will be included as additional data in our qualitative analysis (see Table 1).

**TABLE 1:** Inclusion criteria.

Inclusion criteria for posts	Inclusion criteria for comments
Made on select moderated subreddits	Made on select moderated subreddits
Written in English	Written in English
User age specified (12–18)	No personal identifiers
No personal identifiers	Relate to anxiety
Relate to anxiety	Reacts to the original post by providing mental health‐related information/advice
Specifies psychological experiences of anxiety AND/OR shares an experience of undergoing treatment	Reacts to the original post by providing an emotional reaction (e.g., reassurance or doubt)
Minimum length 600 characters	Minimum length 200 characters

## 3 Results

**FIGURE 1:**
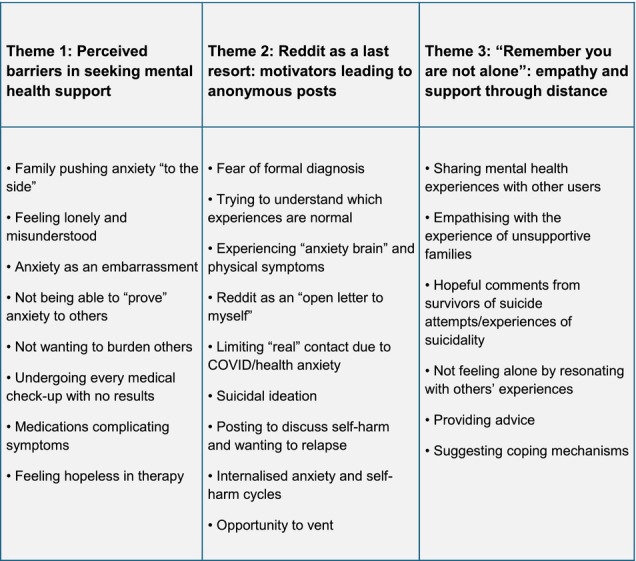
Mind map.

Our thematic analysis identified three themes: *Perceived barriers in seeking mental health support* (Theme 1), *Reddit as a last resort: motivators leading to anonymous posts* (Theme 2), and ‘Remember you are not alone’*: empathy and support through distance* (Theme 3). See Figure 1 for a detailed thematic mind map containing codes.

Retrieved data from T1 revealed that many adolescents feel let down by professional support (e.g., counselling and GP). In addition, many adolescents are also afraid of being stigmatised or rejected by their family members and friends. As a result, many young people turn to anonymous online spaces to seek alternative treatment forms and information about coping mechanisms. T2 demonstrates the reasons why young people turn to the virtual space when it comes to mental health distress. Asking anonymous users for psychological (and, at times, medical) advice, posting as a way of narrating one's experiences, and sharing when anxiety feels like it is all too much (particularly in cases of suicidal ideation) have emerged as key motivators for posting about anxiety on Reddit. Our final T3 reflects on the comments received from the original posters where anxiety was discussed. In our analysis, there was an overwhelmingly positive reaction to users conveying mental health distress and experiences of anxiety on Reddit. This may be due to retrieving posts from subreddits that explicitly focus on mental health, and, as such, they may be frequented by users who have a supportive approach toward individuals experiencing similar psychological struggles to those of their own.

### 3.1 Theme 1: Perceived Barriers in Seeking Mental Health Support

#### 3.1.1 Feeling Let Down by Professional Support

There were users who described their symptoms as chronic or ongoing, where they had either historically received treatment or were currently in treatment and receiving professional support. However, users pointed out that, particularly when it comes to more severe cases of anxiety, professional support is experienced as limited and not able to meet their needs. Users noted that medication can complicate already difficult physical symptoms. Some users had sought help via alternative treatment pathways or were looking online for advice on coping mechanisms and breathing exercises (this includes Reddit).

User no. 25 (age 15): In the past 3 years, I have experienced every medical checkup possible, been to the ER a number of times and, in the end, was diagnosed with generalised anxiety and potential PTSD. But even with my diagnoses, I still feel like I'm constantly on the verge of death. I've been to therapy and a psychiatrist but nothing helped. The medication I've been prescribed ‐ no matter how strong ‐ did not work for me or made my symptoms worse.

#### 3.1.2 Feeling or Fear of Being Stigmatised, Rejected or Shamed

Users described feeling lonely and misunderstood, especially in their relationships with family and friends. Users note that some of the anxiety symptoms can be thought of as normal or not evident, and therefore difficult to prove; this often leads to poor communication. Newly established relationships, such as those with partners and friends, are also experienced as difficult. Respondents noted experiencing social anxiety to be up to social standards.

User no. 28 (age 18): I am on medication and told my boyfriend how happy I was because it finally started to work. He told me that he expected that my anxiety would be completely gone by now. I said, ‘Where would it go? It's not a cold!’ I told him he was unfair and should support me, but he said that ‘we are both adults’ and that I should ‘figure out something as basic as this’ by myself.

User no. 7 (age 18): I would like to see a doctor so that I can get medication for my anxiety. But I'm afraid of my parents, I'm afraid of what they will think of me and do with me when they find out that I have anxiety and depression.

Common concerns were made to the health and safety of a family member. Part of their need to post on this platform appeared to be because they felt that they could not speak to their family because they did not want to add to the burden of their family members' psychological difficulties.

User 11 (age 16): I haven't told my Mum about my anxiety or recent suicidal thoughts. I would like to tell her about what I'm going through, but she is going through so much now herself. I hear her crying over the phone sometimes. I can't break her heart again. She was so hurt last time she found out that I thought of hurting myself. If anyone out there has some advice for me, I'd greatly appreciate it.

### 3.2 Theme 2: Reddit as a Last Resort: Motivators Leading to Anonymous Posts

#### 3.2.1 Posting for Advice and/or Support

Users often end their posts by asking questions about their symptoms or seeking psychological and medical advice. Many posts include descriptions of experiences and symptoms that are frequently presented with the question.

User 57 (age 15): My symptoms include overwhelming feelings of guilt and regret, constant fidgeting, headaches, chronic worry, and mood swings. Is this normal?

#### 3.2.2 Posting as a Way of Making Sense of One's Experiences

Some users described continuous physical symptoms. Other users noted that COVID intensified their experiences of anxiety such as limited social interactions and developing health anxiety.

User 7 (age 14): I thought I was doing better but for the past two hours I've been pacing in the kitchen and clawing at my arms until the skin broke. But now I just found out that my neighbour has COVID and now I … I don't know what to do, my stupid brain just. won't. shut. up.

User 30 (age 15): Stress and anxiety are killing me. Since COVID started I haven't gone to school because our area has been labelled as a hot spot for COVID. I haven't seen anyone in over a year and I just don't know that I can handle seeing many people suddenly after lockdown.

There were young people who used the subreddit as a diary to describe their current circumstances or as an ‘open letter’ to themselves: describing the situation and making counterarguments with themselves on what they need to do moving forward.

User 12 (age 16): God, I'm so tired. I don't why but it really hurts. It's really cold today, but because of my anxiety, I keep getting super hot. I live in a really big desert, and anything could go wrong at any point. Fire, whole mesa lights up. Or a bad storm, tons of mud, difficult to get through. We hear gunshots a lot here. It could be wild animals. Could be unfamiliar people. The best thing? There's no civilisation. I could probably get lost super easy. There's a lot of vultures and coyotes too. I'd be a nice snack for them. Or i could freeze to death. Or get severely overheated in the summer.

#### 3.2.3 Posting As a Last Resort When Feeling Like It Is All Too Much: Suicidal Ideation

There were users, who shared suicidal thoughts and attempts, which referred to cycles of internalised anxiety and self‐harm; their narrative often alluded to the fact that their experience of anxiety goes beyond symptoms, as it has become a part of their identity and way of perceiving the self and the world. Several users have also disclosed deeper personal experiences, including instances of physical and sexual abuse, assault, and trauma.

User 72 (age 17): I don't really remember what it feels like not to be anxious. It's like the fear is part of me now. Some days it gets so heavy that I start thinking it would be easier if I wasn't here anymore. My brother used to touch me when I was 5, it is no wonder I feel like this.

### 3.3 Theme 3: ‘Remember You Are Not Alone’: Empathy and Support Through Distance

In analysing responses to original posts, we found largely positive reactions to users conveying mental health distress and experiences of anxiety. This may be due to our study retrieving posts from subreddits that explicitly focus on mental health, and, as such, they may be frequented by users who have a supportive approach toward individuals experiencing similar psychological struggles to those of their own. Indeed, positive encouragement, support and understanding have been observed in wider contexts. For instance, commentators were keen to share and empathise with the specific experience of unsupportive environments, particularly unsupportive family members.

Responder 6: Hey sis, I just wanna tell you that you are worth it and you don't have to consider your blood family as your real family. You can one day escape your unsupportive parents and find a chosen family that will love you for who you truly are. One day it will get better, please keep believing that.

Other responders shared that reading vulnerable posts depicting the intense experience of anxiety as well as other mental health issues gave them hope in that they are not alone. Responders would convey that the user's symptoms are a normal response to their experiences they described emphasising the need for symptoms not to be pathologized.

Responder 16: Hey man, I'm in a very similar situation as you. Honestly, reading your post made me feel a little bit less alone knowing that there are other people out there in the same situation as me. You're not alone and life will get better, I promise you. You have a long life ahead of you and you're going to get better. Time heals.

Responders also sought to give advice and suggestions for adaptive coping mechanisms. In these instances, responder's shared similar experiences and offered suggestions which were helpful for them. This meant that the responders could offer peer support through their lived experiences.

Responder 25: Try enjoying your own company as well. Become less reliant on other people's company to fulfil you. There was a time when I only had one friend, now I have quite a few friends. I became less bothered about other people and enjoyed being by myself.

## 4 Discussion

This study explored how adolescents use the online social media platform Reddit while experiencing symptoms of anxiety. Our findings reflect a fear commonly felt by adolescents in relaying their experiences to family members and professionals (Fusar‐Poli et al. 2024). The reasons for this varied: adolescents reported fears of stigma and/or rejection by their family members or friends; shared concerns that their mental health experiences would not be seen as sufficiently serious, which may pose unrealistic expectations for getting better and instigate shame when recovery is challenging. This aligns with a systematic review where 76% of the studies, reported that young people experienced or anticipated stigma and embarrassment (Radez et al. 2021). Lastly, some of the adolescents experienced dissatisfaction with mental health treatment, which created further stumbling blocks in seeking professional help. Collectively, these experiences act as strong motivators for seeking advice in anonymous online spaces.

Consistent with research on communication with weak ties (i.e., relationships involving low emotional intensity and limited intimacy), people experiencing mental health distress often seek out and sometimes prefer everyday weak ties, such as interactions with neighbours or acquaintances, over interactions with strong ties, such as family members (Moreton et al. 2023). These weak ties and strangers are widely available, particularly in technologically mediated spheres such as social media. In addition, weak ties are frequently able to offer support when a stressful event is occurring by sharing information and advice based on one's own experiences, which is particularly important for those suffering from mental health distress and trauma (Gottlieb and Bergen 2010). Although this study did not investigate adolescents' relationships with weak ties, given our focus on virtual interactions with anonymous users, our findings emphasise that, for young people, interactions with strangers who have similar mental health experiences can provide more support than interactions with family members. This is particularly true for young people who are experiencing family unavailability, lack of support, mental health shame (Moreton et al. 2023). Our findings also indicate that some young people avoid sharing their distress with family members who are undergoing their own mental health challenges.

While there is a wide availability of research on support from weak ties, few studies are available on support from anonymous users online in the context of mental health distress (Dixon‐Ward and Chan 2022). Our findings, despite being limited to a specific social media platform, and anxiety as a specific mental health experience, indicate that online help‐seeking behaviours among young people are a reality. Indeed, today's youth embodies the term digital natives (Prensky 2001) fluent in the digital language of technology. Therefore, it is important to differentiate between adolescents and adults when trying to understand help‐seeking behaviours and patterns. Research indicates that online support appears more acceptable and accessible for younger adults and adolescents in particular (Seward and Harris 2016). Despite this, there is little research investigating how this type of online support might impact young people. Our study sheds light on the positive side of sharing experiences of anxiety on mental health, with other users offering support, advice, and empathic responses. A study conducted by Dixon‐Ward and Chan (2022) on attitudes toward depression online, on the other hand, has found that some users can be less supportive, enforcing barriers to help‐seeking. It is therefore important to understand how these digital reactions to mental health issues are formed, and how they might impact further mental health help‐seeking among young people, both in face‐to‐face as well as online contexts. Above all, our research findings emphasise the increasing importance of the internet during mental health distress, noting that, amidst various virtual dangers (e.g., cyberbullying), it may also provide comfort for those young people who feel alone or let down.

This provision of comfort is one coping mechanism the findings of our analysis observe, a strategy individuals use to manage their overwhelming feelings. Emotional disclosure, a well‐researched method of coping (Muris et al. 2002; Niles et al. 2014), is one of the ways that social media can provide comfort to individuals. By sharing their feelings, adolescents can connect with others who may experience similar challenges, receive support and validation, and alleviate feelings of isolation, thereby improving overall mental health. Following mental health forums, participating in online support groups, and information‐seeking on social media are other coping mechanisms that social media platforms can offer to manage anxiety.

Although our paper did not explicitly focus on suicide and experiences of suicidality among adolescents online, the data revealed that young people can use social media to express these feelings. In this instance, social media platforms that do not prevent users from sharing experiences pertaining to suicidal thoughts and/or suicide attempts, can be particularly important for young people who are looking to document internalised cycles of anxiety, and to hear from people who share similar mental health experiences. The literature exploring the correlation between social media, internet use and suicide attempts is conflicting. However, a systematic review by Sedgwick et al. (2019) highlighted that problematic use of social media increased suicide attempts in seven studies. Findings from our study indicate that young people who suffer from mental health issues and have little to no social support are particularly at risk for suicide‐related behaviours. Loneliness, past trauma, and co‐morbid mental health issues have also been highlighted as risk factors for suicidality by other studies (Jha et al. 2023; Robinson et al. 2016).

A significant strength of this study is that, through internet‐mediated research, it captured experiences from a traditionally underrepresented group. However, the limitations of our anonymous sample, mean that demographic information cannot be confirmed. Additionally, our study focused on mental health subreddits; it is possible that our findings reflect a positive attitude toward young people's mental health distress that is specific to the subreddit community but less likely across different social media platforms. Finally, since our study analyzed posts written in the English language, it is difficult to make any culturally informed observations. Future internet‐mediated research on adolescents should expand the methods to ensure a homogeneous sample is maintained.

## 5 Conclusions

These findings highlight that technology, as a vehicle for communicating psychological needs for young people, should not be disregarded by mental health services. Social media sites such as Reddit offered adolescents a platform to express concerns related to anxiety. Such concerns often embodied feelings of hopelessness and extreme worry, in addition to anxiety‐related physical symptoms. Nevertheless, those responding to posts on mental health subreddits consistently answered with reassurance, validation, and experiential knowledge. Despite online posts conveying an array of feelings centered around helplessness and hopelessness, they also convey a sense of hope in recovery for the young person. Their motivation and action were taken to reach out in this way, even those conveying thoughts of suicide, indicating an ability to explore alternative coping strategies.

## 6 Relevance for Clinical Practice

It is important to understand the role social media plays in young people's emotional regulation, identity, and peer support. Awareness of online influences helps inform tailored, empathetic care. An open dialogue about online experiences should be encouraged to support safe coping strategies and strengthen therapeutic relationships. Professionals could also explore the potential therapeutic effect of weak ties online in the role of an administrator through technology‐mediated platforms. However, intervention design and research measuring practicalities, safeguarding, and patient outcomes are required before implementation.

## Funding

The authors have nothing to report.

## Conflicts of Interest

The authors declare no conflicts of interest.

## Data Availability

The data that support the findings of this study are available from the corresponding author upon reasonable request.
